# SonoElastoColposcopy: A New Tool for Cervical Dysplasia Assessment

**DOI:** 10.3390/diagnostics15070806

**Published:** 2025-03-22

**Authors:** José Antonio Sainz-Bueno, Cristina Fernández-Conde de Paz, Ainhoa Holgado, José María Romo, Teresa Reyes-Sánchez, Fernando Fernández-Palacín, José Antonio García-Mejido

**Affiliations:** 1 Department of Surgery, Faculty of Medicine, University of Seville and Gynecology, 41004 Sevilla, Spain; jsainz@us.es; 2 Andalusian Public Foundation for the Management of Health Research in Seville (FISEVI), 41009 Seville, Spain; cristinafconde@hotmail.com (C.F.-C.d.P.); ainhoa.holgado.sspa@juntadeandalucia.es (A.H.); josem.romo.sspa@juntadeandalucia.es (J.M.R.); terreysan@gmail.com (T.R.-S.); 3 Department of Statistics and Operational Research, University of Cadiz, 11003 Cadiz, Spain; fernando.fernandez@uca.es

**Keywords:** elastography, cervix uteri, cervical dysplasia

## Abstract

**Background/Objectives:** Up to 30% of cervical dysplastic lesions are missed by colposcopy alone. We performed a comparative evaluation of the diagnostic capacity for identifying cervical dysplastic lesions between shear wave elastography (SWE) of the endocervix and exocervix, defined as SonoElastoColposcopy (SEC), and colposcopy. **Methods:** A prospective observational study was conducted in 84 patients indicated for cervical conization surgery (presence of cervical intraepithelial neoplasia 2 or 3 (CIN-2 or 3), adenocarcinoma in situ (AIS), or high-grade suspicious lesions). All patients underwent colposcopy with lesion identification and biopsy, and SEC and SWE evaluation of the endocervix and exocervix with measurement of lesion stiffness (KPa). Cervical lesions identified by colposcopy or SEC were localized in quadrants, and a comparative evaluation of the diagnostic capacity of both techniques was performed in relation to the anatomical pathology of the cone biopsy. **Results:** A total of 82 women were evaluated (two cases were lost). The mean age was 38.84 ± 8.44 years. Colposcopy was adequate in 95.12% of cases. In SEC, we observed an elasticity in the lesion area of 105.42 ± 36.32 KPa compared to 19.98 ± 9.29 KPa (*p* < 0.0001) in the healthy area of the exocervix. In the endocervix, the results were 109.8 ± 40.86 KPa versus 18.5 ± 9.07 KPa (*p* < 0.0001), respectively. The concordance for colposcopy was 0.456 compared to 0.815 (*p* < 0.05) for SEC. **Conclusions:** SEC demonstrates a better ability to identify the area of cervical dysplastic lesions than colposcopy.

## 1. Introduction

Cervical cancer is one of the most common cancers in women worldwide [1,2]. Each year, more than half a million women are diagnosed with cervical cancer and the disease causes more than 300,000 deaths globally [1,2]. In recent decades, the incidence of cervical cancer has decreased in developed countries due to the implementation of screening programs [3,4]. High-grade squamous intraepithelial lesions (H-SILs) and cervical cancer are caused by persistent human papillomavirus (HPV) infection [5,6]. 

Cytology is the most accessible, feasible, and cost-effective tool for detecting H-SIL and invasive cancer [7,8]. The exact accuracy of Papanicolaou testing is difficult to determine, ranging from 53% to 78% [8]. The combination of colposcopy, cytology, and colposcopically guided biopsy is the gold standard for the diagnosis of H-SIL and cervical cancer [9,10]. While the detection of cervical cancer is highly effective, the management of H-SIL remains a public health challenge [10]. 

After the presence of abnormal cytology, colposcopy is the most cost-effective examination for accurate diagnosis of H-SIL, allowing the examiner to locate potential lesions, assess their severity, and obtain guided biopsies [11,12]. Colposcopy is more effective than conventional or liquid-based Papanicolaou (Pap) smears in detecting H-SIL and is better at distinguishing H-SIL from low-grade squamous lesions (L-SIL) than L-SIL from a normal cervix [11,12]. 

Nonetheless, colposcopy remains of clinical interest. It has a wide range of sensitivity and specificity for detecting H-SIL, ranging from 30 to 90% and 44–97%, respectively, with up to 30% of H-SIL cases not detected by colposcopy alone [12,13,14]. Possible explanations for the wide range of sensitivity and specificity of colposcopy include the experience level of the colposcopists, the type of transformation zone, and the age of patients [13,15,16]. 

Therefore, new methods to detect H-SIL and invasive cancer have been investigated in recent years [13,17]. Sonoelastography is useful in assessing tissue stiffness and has been shown to be useful in liver, breast, and thyroid pathology [17,18]. Previous studies have demonstrated that cervical stiffness, as assessed by elastography, is increased in H-SIL and invasive cancers [19,20]. We now propose the identification of cervical lesions, H-SIL, and invasive cancer by SonoElastoColposcopy (SEC), a new ultrasound technique in which colposcopy is performed with elastography, and we compare its diagnostic capacity with the gold standard: colposcopy. 

## 2. Materials and Methods

### 2.1. Characteristics of the Study Population 

A prospective observational study was conducted in which 84 women indicated for cervical conization were consecutively recruited at a Cervical Pathology consultation between 1 January 2022 and 30 June 2023. Patients were followed up due to abnormal cervical cancer screening results (cytology or HPV test). In addition, they had to meet the following inclusion criteria to participate in the study: they had to be between 18 and 65 years of age, have signed an informed consent form, and meet the criteria for cervical conization due to a biopsy result showing cervical intraepithelial neoplasia 2 (CIN-2), CIN-3, adenocarcinoma in situ (AIS), or a suspected high-grade [21] During the trial, patients underwent colposcopy, cervical ultrasound using shear wave elastography (SWE), known as SEC, and guided cervical biopsy. 

The following information was collected from the patients: age, body mass index (BMI), smoking habits, parity, menopausal status, and cytological findings (normal, L-SIL, H-SIL, carcinoma, atypical squamous cells where H-SIL cannot be excluded or ASC-H, and atypical glandular cells of undetermined significance or AGUS). We also gathered information on the type of colposcopy (adequate or inadequate), transformation zone (type 1, 2, or 3), presence of minor or major changes on colposcopy, histopathological findings on cervical biopsy (exocervix and endocervix), and SEC findings (kPa). 

### 2.2. Clinical Examination

#### 2.2.1. Colposcopic Evaluation 

Indication for colposcopy was based on the criteria of abnormal cytology or persistent HPV infection for more than 1 year. Colposcopy was performed by applying a 5% glacial acetic acid solution for at least 20 s. In cases where adequate staining was not achieved with acetic acid, Lugol’s iodine (Schiller’s test) was used. The colposcopic assessment was scored and classified according to the observed findings, with adequate evaluation considered to be when the cervix was easily discernible and fully visible in all four quadrants, as well as the transformation zone (TZ). The TZ was further classified as type 1 if the TZ was fully visible and exocervical, type 2 if it was partially or completely within the endocervix but fully visible, and type 3 if it was completely within the endocervical canal and, therefore, not visible. The exocervix was subdivided into four quadrants based on clock positions to better localize lesions: upper left quadrant (0–3 o’clock), lower left quadrant (3–6 o’clock), lower right quadrant (6–9 o’clock), and upper right quadrant (9–12 o’clock). 

The colposcopic findings after application of the glacial acetic acid solution were classified as follows: (1) Minor changes when a thin and irregular acetowhite epithelium or a fine mosaic appearance was observed. (2) Major changes when a dense acetowhite epithelium that stains rapidly, with open glandular orifices, punctuation, and coarse mosaic was observed [22]. If a lesion suggestive of dysplasia appeared, a punch biopsy (Kevorkian) of the exocervix was performed. In cases requiring endocervical histopathological examination (cytological lesions with suspected endocervical or glandular components), a Kevorkian fenestrated curette biopsy was performed by inserting it into the endocervical canal and scraping the four cardinal points. The histopathological result of the biopsies was expressed histologically as CIN-1, 2, 3, or AIS [22]. Patients with CIN-2, CIN-3, or AIS, or those with suspected invasive lesions, were indicated for conization [21]. 

#### 2.2.2. Evaluation Using SonoElastoColposcopy 

The SEC study was performed after colposcopy and prior to conization by an experienced operator with more than 10 years’ experience in gynecological ultrasound and previous training in elastography. The operator was blinded to the clinical data, colposcopy results, and cervical biopsy findings. An Aplio i700 ultrasound device (Canon Medical Systems, Tochigi, Japan) with a vaginal probe (11C3 PVT-781VTE) was used. Shear wave elastography (SWE) was performed with the following presets: frequency 4 MHz, tracking 0, and 4 MHz push-pulse elastography tracking at 0.4 fps. The elastography map had to remain stable for at least 3 s before images were acquired. 

**Figure 1:**
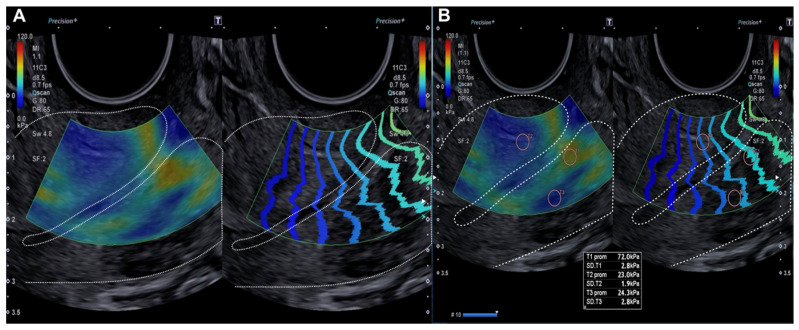
Longitudinal assessment of the cervix (dotted line) by elastography (SonoElastoColposcopy). (**A**) A cervix with low stiffness (blue) and an adequate transmission wave control (in parallel). (**B**) Quantitative assessment of the stiffness measured in Kilopascals (Kpa).

The SEC procedure consisted of a 2D evaluation of the cervix using the vaginal probe, with sufficient ultrasound gel and without applying pressure to the cervix. Initially, the cervix had to occupy three-quarters of the screen in the midsagittal plane before elastography was activated. A 30 mm region of interest (ROI) box was used for the sonographic study, and the elastography velocity map had a scale of 0.5 to 8.5 cm/s, with blue indicating soft or pliable tissue. Overpressure was controlled by ensuring that there was no peripheral red signal on the elastography map and that there were parallel lines on the wave propagation control map (Figure 1). A qualitative assessment of cervical stiffness or elasticity was performed, reporting low or soft stiffness (blue), or high stiffness (red). A quantitative assessment (2 mm circular windows) of cervical stiffness was then performed by taking two measurements in the red areas of interest in the longitudinal section and two measurements in the blue control area, assessing elasticity (kPa). 

**Figure 2:**
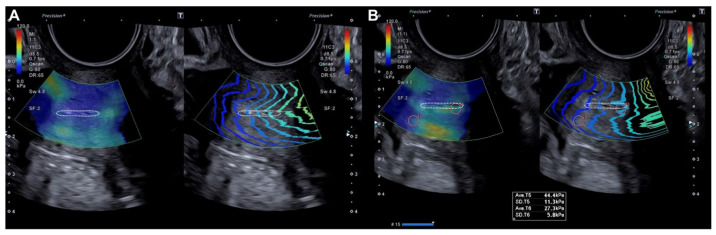
Transverse evaluation of the uterine cervix at the level of the OCE (dotted line) by elastography (SonoElastoColposcopy). (**A**) A cervix with low stiffness (blue) and an adequate transmission wave control (in parallel). (**B**) Quantitative assessment of the stiffness measured in Kilopascals (Kpa).

The probe was subsequently rotated 90º and the external cervical os (ECO) was identified in the transverse section of the cervix using 2D ultrasound. In this transverse location of the ECO, elastography was reactivated and a new qualitative assessment was performed, identifying areas of increased stiffness (red color) (Figure 2). A quantitative assessment of the areas of increased (red) and decreased stiffness (blue) was then performed (Figure 2). Lastly, the locations of the lesions were described according to the model previously used in colposcopy. 

#### 2.2.3. Cervical Conization 

After colposcopic and SEC examination, patients underwent conization using a 20 × 12 mm semicircular diathermy electrosurgical loop in a single incision. In cases with lesions showing deep involvement on previous evaluation, a “cup-shaped” excision of the endocervical canal was performed [21]. After sectioning of the surgical specimen, the base of the surgical bed was coagulated with a 5 mm electrosurgical ball. The surgical sample was referenced with a suture at the 12 o’clock position, and sent for histopathological characterization of the lesion. 

### 2.3. Statistical Analysis 

Statistical analysis was performed using IBM SPSS Statistics version 22 (IBM, Ar-monk, NY, USA). Descriptive analysis was performed using mean and standard deviation for numerical variables and percentages for categorical variables. Numerical variables were compared using a Student’s *t*-test if the data followed a normal distribution (Shapiro–Wilk test), or a Mann–Whitney U test if the data were not normally distributed. Categorical variables were compared using a χ^2^ test or Fisher’s exact test. *p* values < 0.05 were considered statistically significant. Cohen’s kappa coefficient and its 95% confidence interval (CI) were used to assess the agreement between colposcopy and SEC with the histopathological findings of the cervical conization sample. Agreement was considered poor if the kappa coefficient was <0.20, weak if between 0.21 and 0.40, moderate if between 0.41 and 0.60, good if between 0.61 and 0.80, and very good if between 0.81 and 1.00. 

## 3. Results

Of the 84 women initially included, 2 were excluded due to loss to follow-up, leaving a total of 82 patients who completed the study. The general demographic characteristics of the study population, classified according to cytological and histopathological risk, are summarized in Table 1. The mean age of the included population was 38.84 ± 8.44 years. According to age, 56.10% of the population was between 35 and 49 years old. Regarding cytological risk, 50% of patients with low cytological risk were between 20 and 34 years old, whereas 61.66% of patients with high cytological risk were between 35 and 49 years old. The mean BMI of the women enrolled was 23.74 ± 3.52, with 8.54% having a BMI greater than 35. Regarding smoking habits, 48.78% of the study population were smokers. With regard to parity, 31.71% were nulliparous, 36.58% were primiparous, and 31.71% were multiparous. In the low cytological risk group, 45.45% of patients were nulliparous, whereas 43.33% of patients with high cytological risk were nulliparous. Primiparous patients accounted for 41.67% of those with low histopathological risk compared with 35.94% of those with high histopathological risk. 

The cytological characteristics, colposcopic findings, and histopathological results of the patients, classified by cytological and histopathological risk, are shown in Table 2. The cytological diagnoses were normal in 7.32%, L-SIL in 19.51%, H-SIL in 31.71%, carcinoma in 4.88%, ASC-H in 13.41%, and AGUS in 23.17%. Colposcopy was adequate in 95.12% of all cases, 95% of patients with high cytological risk, and 95.31% of patients with high histopathological risk. Only 4.88% of the population had a transformation zone (TZ) located entirely within the endocervical canal (type 3). The vast majority of the population had minor colposcopic changes (43.90%) or major colposcopic changes (48.78%). Minor colposcopic changes were more common in patients with low cytological risk (72.73%) and low histopathological risk (83.33%). Nevertheless, major colposcopic changes were more common in patients with high cytological risk (56.67%) and high histopathological risk (53.13%). Exocervical biopsies showed normal findings in 4.88%, CIN-1 in 9.76%, CIN-2 in 35.36%, CIN-3 in 42.68%, and no result in 7.32%. CIN-1 lesions had low-risk cytology in 31.82% of cases, and CIN-2 and CIN-3 lesions had high-risk cytology in 40% and 56.66% of cases, respectively. A total of 29 endocervical biopsies were performed, of which 20.73% were normal, 1.22% were CIN-1 or CIN-2, 7.32% were CIN-3, and 4.88% had no histopathological result. 

When elasticity was compared quantitatively using SEC between dysplastic cervical lesions and healthy tissue, significant statistical differences were observed (Table 3). The elasticity of the exocervix without lesions was 19.98 ± 9.29 kPa compared to 105.42 ± 36.32 kPa in the dysplastic lesion (*p* < 0.0001). Similarly, in the endocervix, elasticity without lesions was 18.5 ± 9.07 kPa compared to 109.8 ± 40.86 kPa with a dysplastic lesion (*p* < 0.0001) (Table 3). 

**Figure 3:**
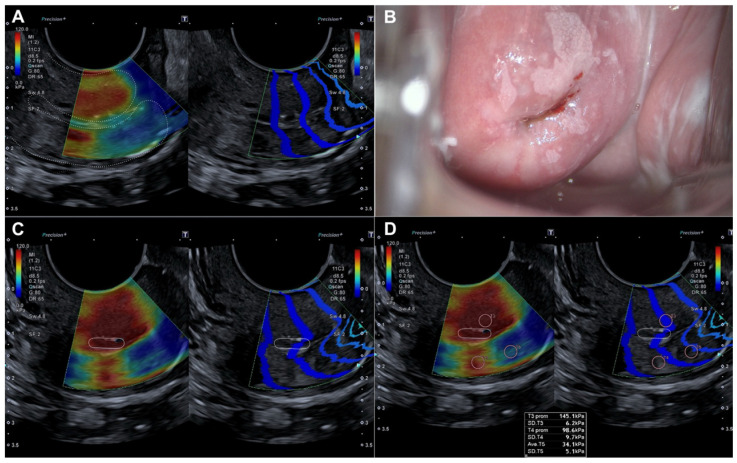
Evaluation by elastography (SonoElastoColposcopy) and colposcopy. (**A**) Longitudinal section of the uterine cervix (dotted line) showing an endocervical dysplastic lesion in red. (**B**) Colposcopic appearance of the dysplastic lesion of the ectocervix at 12 and 6 o’clock. (**C**) Cross-section of the ectocervix by SEC (dotted line) in red showing a dysplastic lesion of the ectocervix at 12 and 6 o’clock. (**D**) Cross-section of the ectocervix by SEC (dotted line) in red showing a dysplastic lesion of the ectocervix at 12 and 6 o’clock, with quantitative evaluation of the dysplastic lesion in KPa.

Histopathological evaluation of the 82 conization specimens, which were used as the gold standard, was performed to locate the lesion in the four quadrants of the cervix as previously divided. When comparing lesion localization in the histopathological study of the surgical sample with the localization shown by colposcopy and SEC, we observed a moderate agreement with colposcopy (0.456 (*p* < 0.05)) compared to a very good agreement with SEC (0.815 (*p* < 0.05)) (Table 4). Figure 3 illustrates how a dysplastic lesion is visualized in both the endocervix and exocervix, and compared with colposcopy. 

## 4. Discussion

We observed that areas of the cervix affected by dysplastic lesions or cancer exhibit greater stiffness as evaluated by elastography. These are represented as areas of rigid tissue (in this case, red), with a significant difference in kPa at the exocervical level of 105.42 ± 36.32 versus 19.98 ± 9.29 (*p* < 0.0001), and at the endocervical level of 109.8 ± 40.86 versus 18.5 ± 9.07 (*p* < 0.0001), when compared to healthy cervical tissue. These findings are consistent with the existing literature on evaluation of the cervix using strain elastography with color scores or shear wave elastography (m/s or kPa) [23]. In previous studies by our group, we observed changes in cervical stiffness in relation to parity and age [19], with greater stiffness in multiparous women (4.41 vs. 2.67 m/s, *p* < 0.002, multiparous vs. nulliparous) and in women older than 50 years (5.05 vs. 2.75 m/s, *p* < 0.008 between patients aged 20 and 50 years). Other authors, such as O’Hara et al. [24], confirm these changes. Although there are changes in cervical stiffness with parity and age, they are not as pronounced as those seen in dysplastic lesions and cervical cancer. Lu et al. [25], Bakay et al. [26], and Xie et al. [27] clearly identify stiffer, color-change regions in the cervix with both benign and malignant pathology using strain elastography. Machanda et al. [28] reported a mean stiffness of healthy cervices assessed by SWE of 18.90 ± 4.22 kPa, a result consistent with our findings. Lu et al. [25] and Lui et al. [29] reported greater differences in stiffness between healthy and malignant cervixes. Lui et al. [29] reported a mean stiffness of 2.86 ± 0.23 vs. 4.91 ± 1.12 m/s between healthy cervices and malignant lesions. This author proposed a cut-off point for malignancy of 3.95 m/s, with a sensitivity and specificity of 79% and 75%, respectively. A previous study by our group [20] was the first to evaluate the stiffness difference between a healthy cervix and one with cervical dysplasia using SWE, observing respective stiffness values of 3.0 m/s (34.5 kPa) vs. 4.1 m/s (58.6 kPa) (*p* < 0.001) and identifying a cut-off for cervical dysplastic pathology at 3.25 m/s (sensitivity 62.5%, specificity 75.5%). The current data confirm these previous findings and show that these differences are observed at both the exocervical and endocervical levels when there is involvement at these sites. 

All these data, combined with the information provided by Dudea-Simon et al. [23] in a systematic review, which indicates that elastography has an intraobserver and interobserver intraclass correlation coefficient (ICC) of 0.96 and 0.94, respectively, present elastography, specifically in its SWE form, as a useful tool for the diagnosis and management of dysplastic lesions and cervical cancer. 

In this study, we have taken a further step in the evaluation of cervical dysplastic pathology by presenting a novel technique for evaluation of the uterine cervix using SWE, which we have defined as SEC. In this approach, we perform an initial longitudinal evaluation of the endocervix using SWE and identify differences between cervices with endocervical dysplastic lesions and those without (109.8 kPa vs. 18.5 kPa, *p* < 0.0001). We have taken a further step in the evaluation of cervical dysplastic pathology by presenting a novel technique for evaluating the cervix using SWE, which we have defined as SEC. In this approach, we perform an initial longitudinal evaluation of the endocervix using SWE and identify differences between cervices with and without endocervical dysplastic lesions (109.8 kPa vs. 18.5 kPa, *p* < 0.0001). Next, at the exocervical level, we perform a transverse evaluation of the cervix, divided into four quadrants, using SWE to identify and evaluate regions of higher stiffness (in our case, red) versus those of lower stiffness (blue). This method mirrors colposcopy but with SWE ultrasound. As a result of this new SEC technique, and by performing a comparative evaluation with colposcopy for locating dysplastic lesions in the cervix, we achieved a Kappa concordance index of 0.815 for SEC compared to 0.456 for colposcopy. While colposcopy has limitations in locating dysplastic lesions, with up to 30% mislocalization of high-grade dysplastic lesions [12,13,14], we now demonstrate that this new technique, SEC, can be a valuable tool in the management of cervical dysplastic lesions. 

We believe that this new technique may improve outcomes in the identification of cervical dysplastic lesions as it can assess the entire length of the endocervix, which can be limited by colposcopy, especially when evaluating the transformation zone, which is sometimes challenging. Furthermore, given its adequate concordance index, it seems possible to minimize the differences between colposcopy performed by an expert and that performed by a novice, although this should be demonstrated in future studies. All these findings make us optimistic about the possible future use of SEC in cervical dysplastic pathology, and to help colposcopy reduce the 30% of unidentified H-SIL cases, or to provide information in cases of discrepancy between clinical and cytological findings [12,13,14]. 

In addition to the results reported in this study, other authors have used elastography in cervical cancer with good results in assessing tumor size and its extension into the uterus and vagina [26,30]. Within this assessment of tumor extension, studies evaluating the stiffness of the parametrium measured by elastography stand out, where the technique seems to be extremely useful [30]. Moreover, elastography has been used to predict the response to oncologic treatment in these patients, with promising results [31]. 

### Strengths and Limitations

The main strength of our work is the presentation and evaluation of a new tool, SEC, for the assessment of cervical dysplastic pathology. The main limitations include the inability to evaluate the degree of infiltration into the endocervix of cervical dysplastic lesions by histopathology, and the limitation of subgroup evaluations based on high and low cytologic and pathologic risk due to the limited number of cases evaluated. We also consider as limiting factors the availability of shear wave elastography, and the need for training in the use of elastography and in the interpretation of the data obtained. 

## 5. Conclusions 

SonoElastoColposcopy, a type of shear wave elastography of the endocervix and exocervix, identifies the location of cervical dysplastic lesions more accurately than colposcopy. 

## Figures and Tables

**Table 1:** General characteristics of the included population according to cytological and anatomopathological risk.

	All (*n* = 82)	Low Cytological Risk (*n* = 22)	High Cytological Risk (*n* = 60)	Low Pathological Risk (*n* = 12)	High Pathological Risk (*n* = 64)
	Mean (±SD) or %	Mean (±SD) or %	Mean (±SD) or %	Mean (±SD) or %	Mean (±SD) or %
Age (years)	38.84 (±8.44)	38 (±9)	39 (±9)	38 (±8)	39 (±8)
Age Range (years)					
20–34	27/82 (32.93%)	11/22 (50%)	16/60 (26.67%)	4/12 (33.33%)	22/64 (34.38%)
35–49	46/82 (56.10%)	9/22 (40.9%)	37/60 (61.66%)	7/12 (58.34%)	36/64 (56.24%)
50–65	9/82 (10.97%)	2/22 (9.1%)	7/60 (11.67%)	1/12 (8.33%)	6/64 (9.38%)
BMI	23.74 (±3.52)	23.1 (±3.2)	23.9 (±3.5)	22.9 (±3.8)	24.1 (±3.2)
BMI > 35	7/82 (8.54%)	2/22 (9.10%)	5/60 (8.33%)	2/12 (16.67%)	5/64 (7.81%)
Smoker	40/82 (48.78%)	8/22 (36.36%)	32/60 (53.33%)	3/12 (25%)	34/64 (53.13%)
Parity					
Nulliparous	26/82 (31.71%)	10/22 (45.45%)	16/60 (26.67%)	3/12 (25%)	21/64 (32.81%)
Primipara	30/82 (36.58%)	4/22 (18.19%)	26/60 (43.33%)	5/12 (41.67%)	23/64 (35.94%)
Multiparous	26/82 (31.71%)	8/22 (36.36%)	18/60 (30%)	4/12 (33.33%)	20/64 (31.25%)
Menopausal State	4/82 (4.88%)	1/22 (4.55%)	3/60 (5%)	0	4/64 (6.25%)

**Table 2:** Cytological characteristics, colposcopic findings, and pathological results of patients according to cytological and pathological risk.

	All (*n* = 82)	Low Cytological Risk (*n* = 22)	High Cytological Risk (*n* = 60)	Low Pathological Risk (*n* = 12)	High Pathological Risk (*n* = 64)
	Mean (±SD) or %	Mean (±SD) or %	Mean (±SD) or %	Mean (±SD) or %	Mean (±SD) or %
Cytology					
Normal	6/82 (7.32%)	6/22 (27.27%)		5/12 (41.67%)	
L-SIL	16/82 (19.51)	16/22 (72.73%)		7/12 (58.33%)	
H-SIL	26/82 (31.71%)		26/60 (43.33%)		35/64 (54.69%)
Carcinoma	4/82 (4.88%)		4/60 (6.67%)		4/64 (6.25%)
ASC-H	11/82 (13.41%)		11/60 (18.33%)		10/64 (15.62%)
AGUS	19//82 (23.17%)		19/60 (31.66%)		15/64 (23.44%)
Type of colposcopy					
Adequate	78/82 (95.12%)	20/22 (90.91%)	57/60 (95%)	11/12 (91.77%)	61/64 (95.31%)
Inadequate	4/82 (4.88%)	2/22 (9.09%)	3/60 (5%)	1/12 (8.33%)	3/64 (4.69%)
Transformation zone					
Type 1	35/82 (42.68%)	10/22 (45.45%)	25/60 (41.67%)	6/12 (50%)	27/64 (42.19%)
Type 2	43/82 (52.44%)	10/22 (45.45%)	33/60 (55%)	5/12 (41.67%)	34/64 (53.13%)
Type 3	4/82 (4.88%)	2/22 (9.1%)	2/60 (3.33%)	1/12 (8.33%)	3/64 (4.68%)
Colposcopic changes					
No changes	4/82 (4.88%)		4/60 (6.67%)		1/64 (1.56%)
Minor changes	36/82 (43.90%)	16/22 (72.73%)	20/60 (33.33%)	10/12 (83.33%)	29/64 (45.31%)
Major changes	40/82 (48.78%)	6/22 (27.27%)	34/60 (56.67%)	2/12 (16.67%)	34/64 (53.13%)
No result	2/82 (2.44%)		2/60 (3.33%)		
Pathological anatomy of exocervical biopsy					
Normal	4/82 (4.88%)	3/22 (13.64%)	1/60 (1.67%)	4/12 (33.33%)	
CIN-I	8/82 (9.76%)	7/22 (31.82%)	1/60 (1.67%)	8/12 (66.66%)	
CIN-II	29/82 (35.36%)	5/22 (22.72%)	24/60 (40%)		29/64 (45.31%)
CIN-III	35/82 (42.68%)	1/22 (4.55%)	34/60 (56.66%)		35/64 (54.69%)
No result	6/82 (7.32%)	6/22 (27.27%)			
Endocervical biopsy	29/82 (35.37%)				
Pathological anatomy of endocervical biopsy					
Normal	17/82 (20.73%)				
CIN-I	1/82 (1.22%)				
CIN-II	1/82 (1.22%)				
CIN-III	6/82 (7.32%)				
No result	4/82 (4.88%)				

**Table 3:** A comparison of quantitative results in SEC in kPa between normal cervical areas and areas with injuries.

	SonoElastoColposcopy (SEC)		
	Normal. Mean (±SD)	Dysplastic Lesion. Mean (±SD)	*p*
Exocervix (kPa)	19.98 (±9.29)	105.42 (±36.32)	*p* < 0.0001
Endocervix (kPa)	18.5 (±9.07)	109.8 (±40.86)	*p* < 0.0001

**Table 4:** The degree of concordance (kappa index) of colposcopy and SEC to locate the lesion in the different quadrants using the location shown in the anatomopathological study as the gold standard.

Location of the Lesion in Pathological Anatomy of Conization	Colposcopy		SonoElastoColposcopy (SEC)	
	(%)	Kappa Index	(%)	Kappa Index
Without lesion (*n* = 6)	1/6 (16.67%)	0.456 (*p* < 0.05)	5/6 (83.33%)	0.815 (*p* < 0.05)
Upper left quadrant (*n* = 16)	13/16 (81.25%)		15/16 (93.75%)	
Lower left quadrant (*n* = 3)	3/3 (100%)		2/3 (66.67%)	
Lower right quadrant (*n* = 26)	8/26 (30.77%)		18/26 (69.23%)	
Upper right quadrant (*n* = 13)	7/13 (53.85%)		12/13 (92.31%)	
Various quadrants (*n* = 18)	14/18 (77.78%)		18/18 (100%)	
All (*n* = 82)	46/82 (56.10%)		70/82 (85.37%)	

## Data Availability

The data presented in this study are available on request from the corresponding author. The data are not publicly available due to Includes sensitive patient data.

## Abbreviations

The following abbreviations are used in this manuscript:

SWE	Shear wave elastography
SEC	SonoElastoColposcopy
CIN	Cervical intraepithelial neoplasia
KPa	Kilopascals
H-SIL	High-grade squamous lesions
L-SIL	Low-grade squamous lesions
BMI	Body mass index
TZ	Transformation zone
ROI	Region of interest
ECO	External cervical os
CI	Confidence interval
